# NMR-based metabolomics in a clinical cohort: deciphering the metabolic characteristics of gout with the dampness-heat syndrome and elucidate the efficacy of Simiao Pill

**DOI:** 10.1186/s13020-025-01289-6

**Published:** 2026-01-21

**Authors:** Yu Hu, Le Yang, Guangli Yan, Ye Sun, Maojie Wang, Ling Kong, Hui Sun, Xueping Zhao, Xinya Zhang, Runyue Huang, Chang Liu, Ying Han, Xijun Wang

**Affiliations:** 1 State Key Laboratory of Integration and Innovation of Classic Formula and Modern Chinese Medicine, Metabolomics Laboratory, Department of Pharmaceutical Analysis, National Chinmedomics Research Center, National TCM Key Laboratory of Serum Pharmacochemistry, Heilongjiang University of Chinese Medicine, Heping Road 24, Harbin, 150040 China; 2 State Key Laboratory of Dampness Syndrome, The Second Affiliated Hospital Guangzhou University of Chinese Medicine, Dade Road 111, Guangzhou, China

**Keywords:** Gout, Dampness-heat syndrome, Targeted metabolomics, Traditional Chinese medicine, Simiao Pill, Nuclear magnetic resonance

## Abstract

**Background:**

Gout is an inflammatory arthritis caused by purine metabolism disorders. The gout with the dampness-heat syndrome (GDHS) is a common Traditional Chinese Medicine (TCM) syndrome in this kind of disease, yet its modern scientific basis remains poorly understood. Simiao Pill (SMP), a classic formula in treating GDHS, has an unclear mechanism of action.

**Methods:**

We conducted a targeted Nuclear Magnetic Resonance (NMR)-based metabolomic analysis on serum and urine samples from 197 GDHS patients and 101 healthy controls. Multiple machine learning algorithms, including support vector machine (SVM), random forest (RF), and least absolute shrinkage and selection operator (LASSO), were employed to identify potential biomarkers for GDHS. The Apriori algorithm was applied to uncover associations between TCM syndrome manifestations and metabolomic biomarkers. A subgroup of 50 GDHS patients received a 4-week SMP treatment, and their metabolomic profiles were compared pre- and post- intervention.

**Results:**

GDHS patients exhibited a significant remodeled metabolome, characterized by disruptions in pyruvate, amino acid metabolism, and energy metabolism. A panel of 12 biomarkers with high diagnostic power was identified. Association rule mining further highlighted triglycerides and glycine as central nodes showing extensive connections to TCM syndromes. SMP intervention significantly reversed the level of 10 biomarkers (e.g., citrate, glycine, lactate), effectively normalizing perturbations in glycolysis/gluconeogenesis, the tricarboxylic acid cycle, and glycine/serine/threonine metabolism, and lipid homeostasis.

**Conclusion:**

This real-world clinical study systematically delineates the metabolic features of GDHS, innovatively linking TCM syndromes to specific metabolic disturbances. It confirms that SMP exerts its therapeutic effects through multi-targeted modulation of the metabolic network. This work provides a new scientific paradigm for the study of "disease-syndrome-treatment" in TCM.

**Graphical Abstract:**

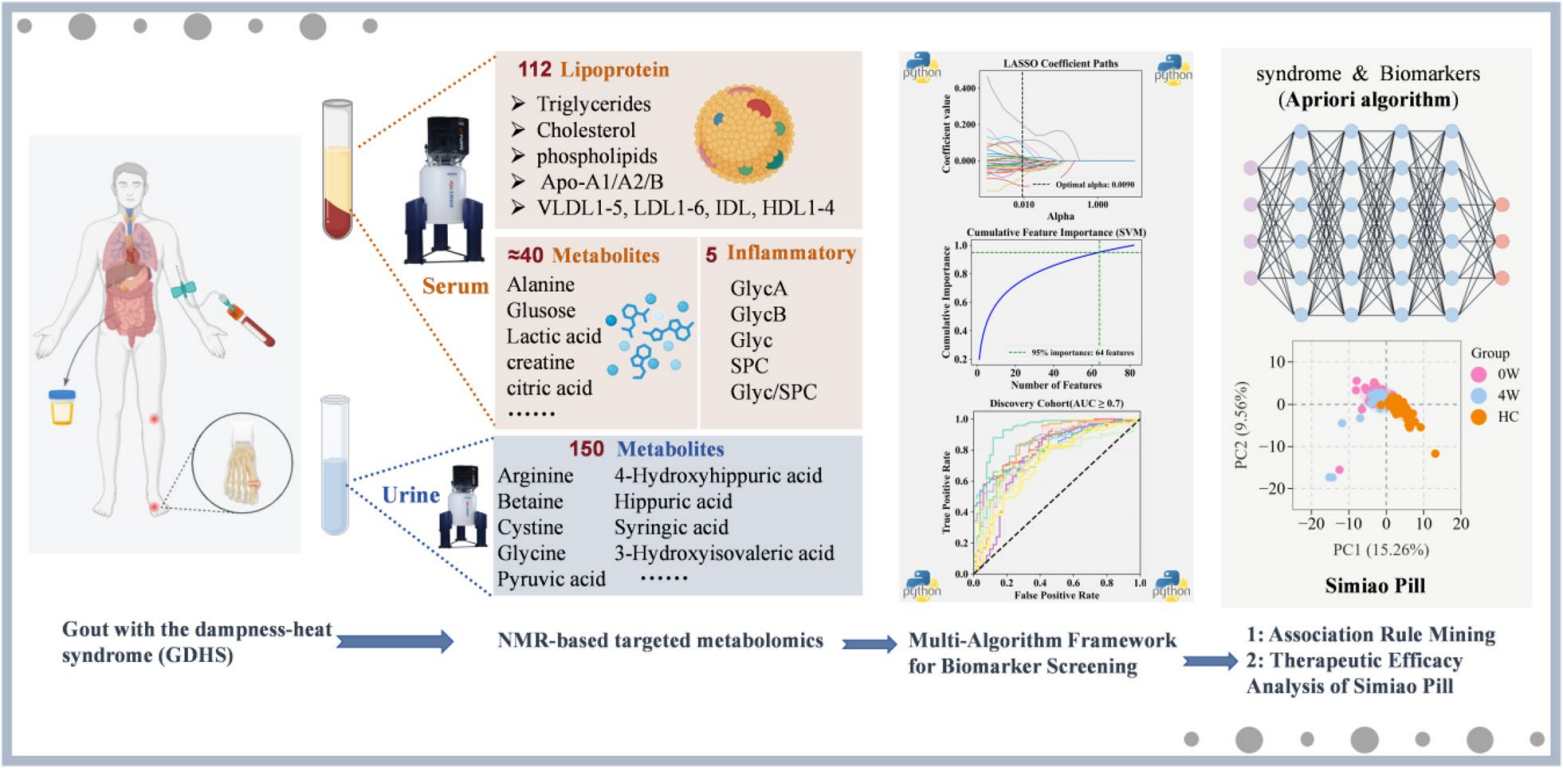

**Supplementary Information:**

The online version contains supplementary material available at 10.1186/s13020-025-01289-6.

## Background

Gout is a common inflammatory arthritis caused by the deposition of monosodium urate crystals in the joints and surrounding tissues. These crystals trigger recurrent inflammatory attacks, characterized by severe joint pain and swelling, most frequently affecting the first metatarsophalangeal joint. As the condition progresses, it can lead to joint deformity and functional impairment [1–3]. Currently, the prevalence of gout is rising and showing a trend toward younger onset, posing a substantial burden on healthcare systems worldwide [4–6]. A recent large-scale study involving 500,000 adults revealed that gout is associated with an increased risk of cardiovascular diseases, diabetes, renal disorders, digestive system diseases, and musculoskeletal conditions [7].

In the theoretical system of traditional Chinese medicine (TCM), gout falls under the category of "Bi Syndrome", which refers to a syndrome characterized by stagnation of Qi and blood, and blockage of meridians by pathogenic factors [8, 9]. Among its various patterns, dampness-heat is one of the most common type. This pattern manifests with intense redness, swelling, heat, and pain in the lower limb joints (such as the knees and ankles), a heavy and fatigued sensation in the limbs, a sticky and greasy feeling in the mouth, and a yellow, greasy tongue coating. It was discussed in detail in Ge Zhi Yu Lun as early as 1347 AD [10, 11]. Notably, gout with the dampness-heat syndrome (GDHS) exhibits a distinct geographical distribution, with the Lingnan region of China being an epicenter of high prevalence. This pattern is attributable to the region's persistent hot, humid climate and a diet rich in high-purine items such as seafood and slow-cooked soups. These factors collectively contribute to a substantially elevated risk of GDHS in the local population, thereby significantly compromising their quality of life.

Despite considerable research progress [12–14], the pathogenesis of GDHS remains incompletely elucidated. Current therapeutic strategies primarily focused on urate-lowering, while effective for symptomatic control, are associated with clinical limitations including an increased risk of cardiovascular mortality, the potential to induce acute gout flares during initial therapy, and possible hepatic and renal damage [15–17]. This suggests that hyperuricemia alone provides an incomplete explanation for the complex pathophysiology of this gout subtype, highlighting the urgent need for mechanistic research and targeted therapeutic development.

Simiao Pill (SMP), which is composed of Rhizoma Atractylodis, Cortex Phellodendri, Radix Achyranthis Bidentatae, and Semen Coicis, is one of the most frequently used formula for GDHS therapy [18–21]. Modern pharmacological studies have revealed that berberine from Cortex Phellodendri exerts anti-inflammatory effects by suppressing the NF-κB/NLRP3 pathway, while also reducing serum uric acid through dual mechanisms: inhibiting xanthine oxidase (XOD) and urate transporters URAT1/GLUT9 to modulate both production and excretion [22–25]. Lactones isolated from Rhizoma Atractylodis have been shown to promote inflammation resolution by modulating the MAPK/STAT signaling pathway and inducing macrophage M2 polarization, thereby contributing to immunoregulation [26, 27]. Phenolic compounds from Semen Coicis inhibit XOD activity, whereas its lipid components facilitate uric acid excretion by remodeling urate transporters, such as downregulating URAT1 and upregulating OAT1/ABCG2 [28, 29]. Total saponins from Radix Achyranthis Bidentatae have been demonstrated to alleviate symptoms of acute gouty arthritis by inhibiting NLRP3 inflammasome assembly and caspase-1 activation, leading to the modulation of downstream inflammatory factors [30]. Collectively, these findings provide a solid theoretical foundation for the use of SMP in treating GDHS. However, research that investigates SMP as an integrated formula remains scarce. Only a limited number of studies have preliminarily reported its potential mechanisms in alleviating gouty arthritis in rat models [31, 32]. The congruence between such animal models and human GDHS, as well as the clinical translatability of the resulting conclusions, warrants further validation.

To bridge the gap between laboratory research and clinical application, this study will innovatively utilize NMR-based targeted metabolomics to analyze serum and urine samples from 298 participants, systematically deciphering the metabolic characteristics of GDHS and exploring their intrinsic relationships with the key syndrome elements of this pattern, thereby providing a scientific basis for its TCM theory. Additionally, by evaluating the capacity of SMP to reverse the identified metabolic disturbances and restore systemic metabolic homeostasis, we seek to clarify its therapeutic mechanisms. Our study seeks to interpret the scientific basis of GDHS at the metabolic level and validate SMP's role in correcting metabolic disorders, thereby offering a new scientific paradigm for integrated "disease-syndrome-treatment" research in TCM.

## Methods

### Clinical subject inclusion

197 GDHS patients and 101 HC individuals who underwent physical examination in Guangdong Provincial Hospital of Chinese Medicine from 2022 to 2024 were selected (Ethical code: BF2020-193-01). Participants were randomly assigned to a discovery cohort and a validation cohort using a random number table method, with each cohort comprising approximately 100 GDHS patients and 50 HC individuals. The discovery cohort was used to identify potential biomarkers, while the validation cohort was utilized to optimize and validate the findings from the discovery cohort.

Inclusion and exclusion criteria for GDHS: (a) Patients who meet the diagnostic criteria for damp syndrome [10, 33], with gout onset in the joints of the lower limbs, were diagnosed as having GDHS. The diagnostic criteria for damp syndrome are shown in Table S1. (b) Exclude individuals with severe cardiac, hepatic, renal, pulmonary, or other organ dysfunction, as well as those with acute or critical illnesses such as malignant tumors. (c) Exclude individuals with psychological or psychiatric disorders. Inclusion and exclusion criteria for HC: (a) The subjects does not meet the diagnostic criteria of GDHS and were diagnosed as healthy by clinicians at Guangdong Provincial Hospital of Chinese Medicine. (b) Exclude those who had recently participated in other drug clinical trials recently.

The study was approved by the Ethics Committee of Guangdong Provincial Hospital of Chinese Medicine, and all participants completed case registration and signed informed consent. This study complied with the Declaration of Helsinki.

### Serum sample collection and preparation

Peripheral blood from all participants was collected using serum separator tubes under standardized protocols. Blood was centrifuged at 1600 × g for 13 min at 4 °C. The supernatant was aliquoted into clean cryovials in 500 μL portions, labeled appropriately, and stored at − 80 °C until further analysis. Dry ice was used during sample transportation to maintain cryogenic conditions.

For ^1^H NMR analysis, frozen serum samples were thawed at room temperature for 30 min. Each sample was mixed with phosphate buffer (75 mM Na_2_HPO_4_, 2 mM NaN_3_, 4.6 mM sodium trimethylsilyl propionate-[2,2,3,3-^2^H_4_] (TSP) in H_2_O/D_2_O 4:1, pH 7.4 ± 0.1) in a 1:1 ratio to a final volume of 600 μL and transferred into 5 mm SampleJet NMR tubes. After brief manual shaking, the samples were stored at 5 °C in the SampleJet autosampler and analyzed within 24 h [34].

### Urine sample collection and preparation

First-morning urine samples were collected from all participants prior to water intake, followed by centrifugation at 6000 rpm for 10 min at 4 °C. The supernatant was aliquoted into sterile cryovials in 1000 μL portions, appropriately labeled, and stored at − 80 °C until analysis. Dry ice was used during all transfers to maintain low-temperature conditions.

For ^1^H NMR analysis, the frozen urine samples were thawed at 20 °C for 30 min. Following established standard operating procedures, 900 μL of urine sample was mixed with 100 μL of phosphate buffer (1.5 M KH_2_PO_4_, 1 mM TSP (sodium trimethylsilyl propionate-[2,2,3,3-^2^H_4_]), 0.13 mM NaN3 in D_2_O, pH 7.4 ± 0.1). A total volume of 600 μL of the resulting mixture was transferred into 5 mm SampleJet NMR tubes for analysis. Prepared samples were maintained at 5 °C in the SampleJet robot and analyzed within 24 h [34].

### ^1^H NMR spectroscopy data acquisition and processing parameters

All NMR experiments were conducted on a 600 MHz Bruker Avance III HD spectrometer equipped with a 5 mm BBI probe and SampleJet cooling system. A pre-analysis quantitative calibration was performed as described previously [34]. All data were acquired using targeted in vitro diagnostic research (IVDr) standard protocols.

For each serum sample, two experiments were completed at 310 K: a ^1^H one-dimensional experiment with solvent presaturation (32 scans, 98 k data points, 30 ppm spectral width) and a Carr − Purcell − Meibo-Gill (CPMG) spin-echo experiment (32 scans, 74 k data points, 20 ppm spectral width). Free induction decay (FID) signals were automatically processed in Bruker TopSpin 4.3.0 for phasing and baseline adjustment. Lipoprotein profiling was performed using the Bruker IVDr Lipoprotein Subclass Analysis (B.I.-LIS) method, which employs a validated prediction algorithm founded on the PLS regression model [35, 36]. 112 parameters including the total lipid analyte phospholipids, triglycerides, cholesterol, free cholesterol, apolipoproteins A1/A2/B100 and the B100/A1 ratio, and their distributions across subclasses of very low-density lipoprotein (VLDL), low-density lipoprotein (LDL), intermediate-density lipoprotein (IDL), and high-density lipoprotein (HDL) subclasses were quantified. Specifically, there were four HDL-subfractions (HDL-1 to HDL-4), six LDL subfractions (LDL-1 to LDL-6), and five VLDL subfractions (VLDL-1 to VLDL-5). Additional analyses quantified 38 low-molecular-weight metabolites (e.g., citrate, lactate, leucine) using the Bruker IVDr quantification in Plasma/Serum(B.I.Quant-PS) method, along with five inflammatory markers (including GlycA and GlycB) via the Bruker IVDr B.I.-PACS method (Table S2).

Urine samples were analyzed at 300 K using the 1D nuclear Overhauser enhancement spectroscopy (NOESY) experiment (32 scans, 65536 data points, a spectral width of 11904.76 Hz). Similar to serum samples, data were processed automatically in TopSpin 4.3.0 and ICON NMR. A total of 150 urinary metabolites were quantified with the Bruker IVDr quantification in urine (B.I.Quant-URe) method (Table S2).

### Biomarker identification and pathway analysis

Due to substantial variations in units and numerical scales among different substances, direct comparison or modeling would result in features with larger magnitudes dominating the analysis. To ensure that the data reflect genuine biological signals rather than artifacts of scale disparity, the data were preprocessed using the following procedures: total sum normalization, base-10 logarithmic transformation, and pareto scaling. The discovery cohort was used for differential analysis, while the validation cohort was employed to verify the results obtained from the discovery cohort.

In this study, multiple unsupervised dimensionality reduction methods—including principal component analysis (PCA), t-distributed stochastic neighbor embedding (t-SNE), and uniform manifold approximation and projection (UMAP)—were employed to visualize high-dimensional data structures and systematically explore heterogeneity within the metabolomic dataset. Significantly differential metabolites/ lipoproteins/ inflammatory factors were identified based on a fold change (FC) threshold of > 1.2 or < 0.83, confirmed by univariate Student’s t-test (two-tailed, p < 0.05). Kyoto Encyclopedia of Genes and Genomes (KEGG) pathway enrichment analysis of these significant metabolites was performed using MetaboAnalyst V6.0 (https://www.metaboanalyst.ca/).

To identify crucial variables from the differential features, we integrated multiple machine learning algorithms, including Least Absolute Shrinkage and Selection Operator (LASSO) regression, Random Forest (RF), and Support Vector Machine (SVM). LASSO regression employs L1 regularization to shrink the coefficients of irrelevant features to zero, enabling automatic feature selection and dimensionality reduction. RF captures complex non-linear relationships and interaction effects by calculating feature importance based on Gini impurity. SVM, combined with Recursive Feature Elimination (RFE), iteratively removes redundant features based on the weight coefficients of its linear kernel. For model training and parameter optimization, all algorithms utilized fivefold cross-validation to ensure robustness. LASSO automatically optimized the regularization parameter using LassoCV. RF optimized key hyperparameters, including the number of estimators, maximum depth, and minimum samples split, via GridSearchCV. The SVM-RFE procedure was implemented using a linear kernel. To minimize algorithm-specific bias, the final set of high-confidence candidate biomarkers was identified as the intersection of features selected by all three methods. Specifically, the selection criteria for each method were as follows: non-zero coefficients in LASSO, and a cumulative importance of at least 95% in both RF and SVM.

Subsequently, univariate logistic regression analysis was performed on the candidate variables using IBM SPSS Statistics 27.0, with a statistical significance threshold set at P < 0.05. The diagnostic performance of these variables was further evaluated by receiver operating characteristic (ROC) curve analysis. Those exhibiting an area under the curve (AUC) of ≥ 0.70 were considered potential biomarkers for GDHS.

### Biomarkers- main syndrome elements association patterns in GDHS

Association rule mining was applied to explore potential associations between biomarkers of GDHS and its principal syndrome elements. Continuous biomarker concentrations were first discretized into high, medium, and low categories using the K-means clustering algorithm, and then integrated with binarized TCM main symptom data to construct a transaction dataset. The Apriori algorithm was then used to extract frequent itemsets under the parameter settings of a minimum support of 0.1 and a minimum lift of 1.5. Association rules with a confidence level above 0.8 were retained as significant associations. All analyses were performed in Python 3.9.13, utilizing the mlxtend library (v.0.23.4) for association rule mining and the scikit-learn package (v.1.6.1) for cluster analysis.

### Assessment of SMP's effects on metabolic disorders in GDHS patients

Fifty randomly selected GDHS patients received SMP treatment for 4 weeks. The medication was administered twice daily, with each dose consisting of two sachets (supervised by Guangdong Provincial Hospital of Traditional Chinese Medicine). Following the intervention, paired serum and urine samples were collected again and subjected to NMR-based metabolomic analysis to evaluate the effects of SMP in reversing the identified biomarkers and restoring dysregulated metabolic pathways.

### Statistical methods

Detailed descriptions of all statistical procedures are provided in the corresponding 'Methods' subsections. In metabolomic analyses, unpaired two-sided Student’s t-tests were applied to assess differences between groups. Data are presented as mean ± standard deviation (SD) or mean ± standard error of the mean (SEM), with a P < 0.05 considered statistically significant. All analyses were carried out using GraphPad Prism (v.10.0), IBM SPSS Statistics 27.0, and Python (v.3.9.13).

## Results

### Physical characteristics of the clinical subjects

**Fig. 1:**
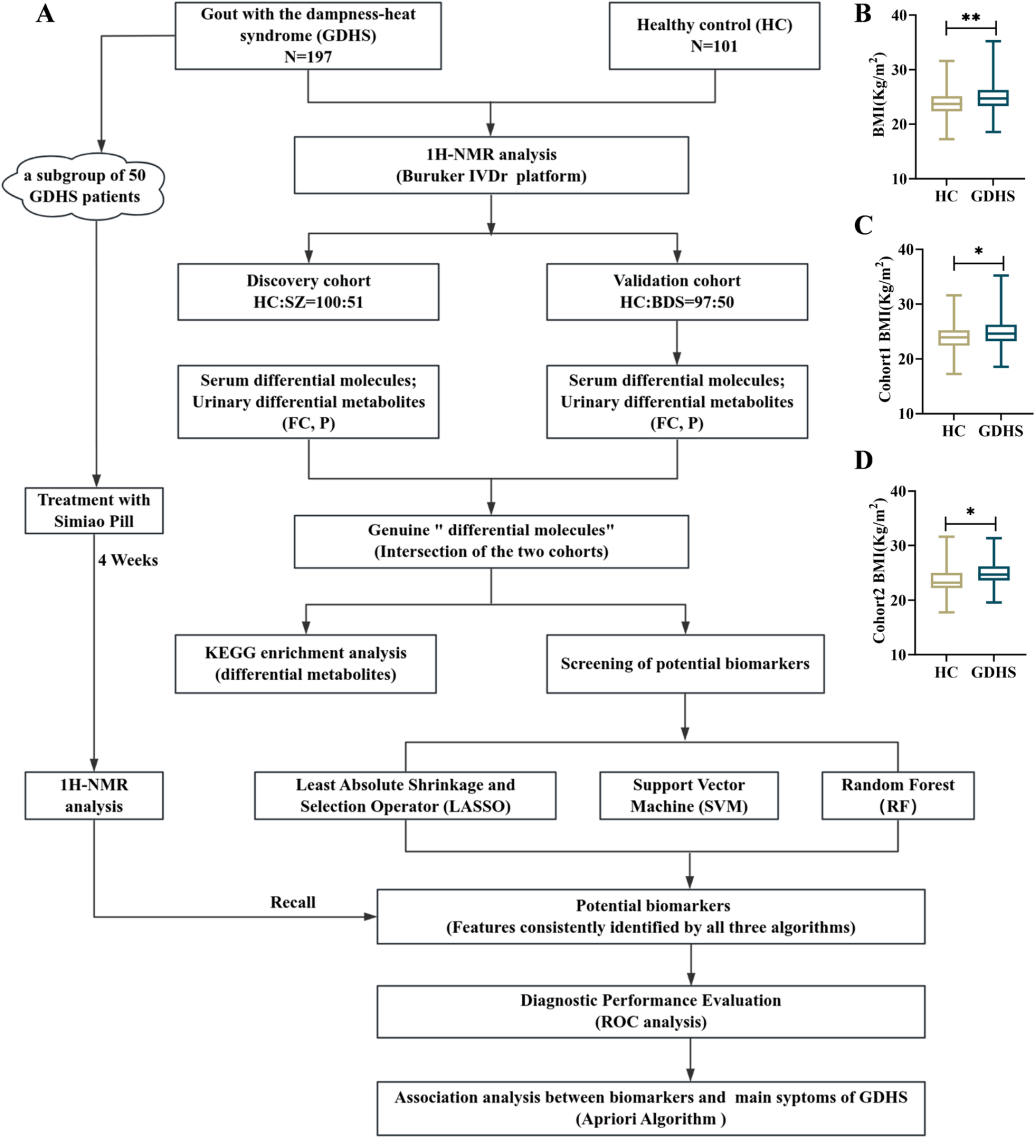
General information of the clinical participants. **A** Study flowchart. **B**–**D** Comparison of body mass index (BMI) between GDHS patients and HC individuals across different cohorts

A total of 298 participants were enrolled in this study, comprising 197 GDHS patients and 101 HC individuals. It is noteworthy that the incidence of GDHS is significantly higher in males than in females [37, 38], a factor that was accordingly considered during the enrollment of HC individuals. Participants were divided into two cohorts: the discovery cohort consisted of 151 subjects (100 GDHS patients, 98.0% male; 51 HC, 96.1% male) and was used for biomarker identification; the validation cohort included 147 subjects (97 GDHS patients, 97.9% male; 50 HC, 96.0% male) and was employed for biomarker verification. Obesity is often associated with insulin resistance (IR), which reduces renal excretion of uric acid and subsequently leads to hyperuricemia [39]. Given that hyperuricemia is a key driver of gout, obesity is thereby an risk factor for the disease [40]. This mechanism may explain why GDHS patients exhibited significantly higher body mass index (BMI) values compared to the HC group. The difference in BMI remained consistent before and after cohort partitioning, indicating that the grouping strategy did not alter the original distribution of this variable. The overall workflow of this study is illustrated in Fig. 1.

### Metabolic reprogramming in the GDHS cohort

**Fig. 2:**
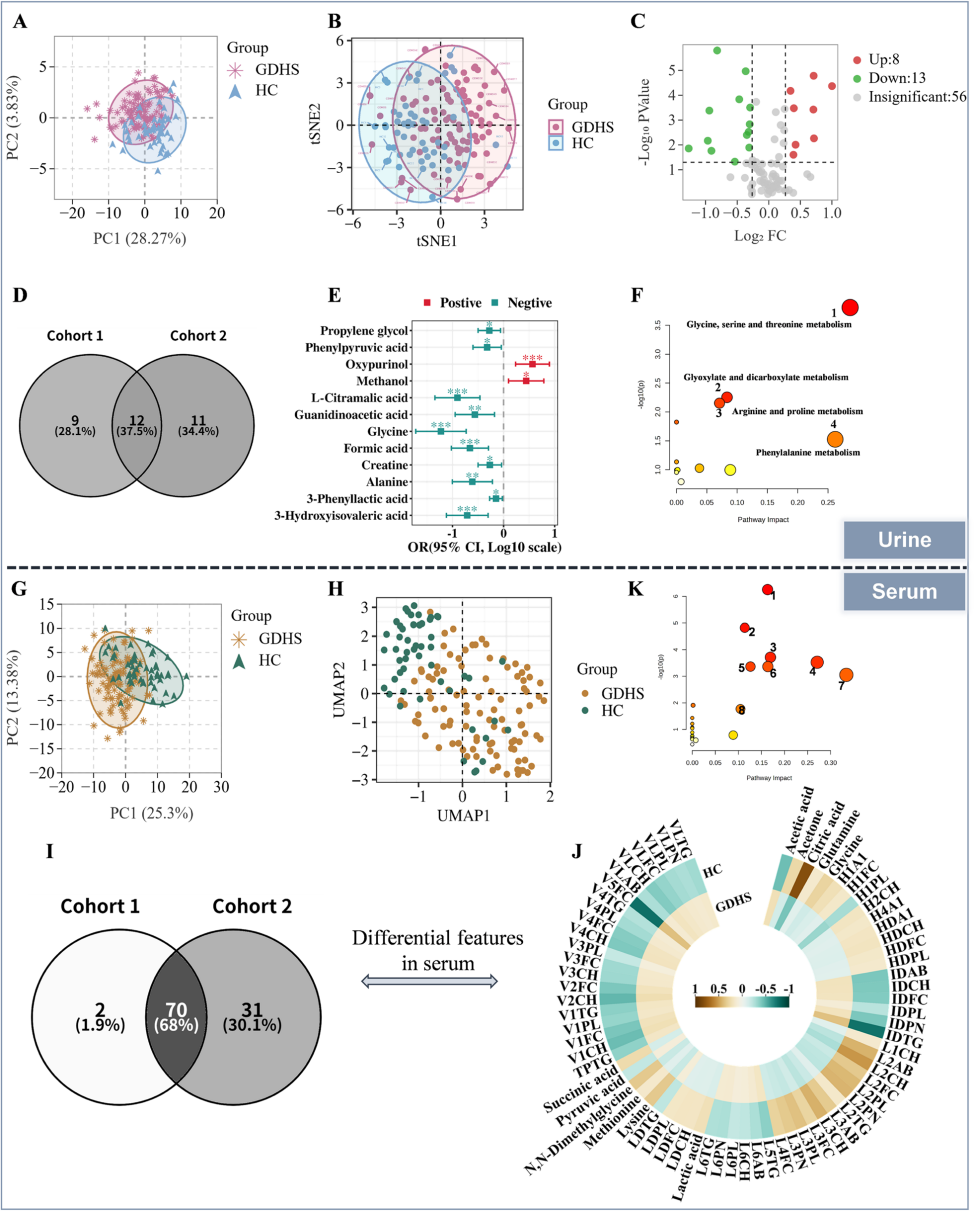
Metabolic reprogramming in GDHS patients (discovery cohort). **A**, **B** PCA and t-SNE of urinary metabolomic data demonstrate distinct separation between the HC group and GDHS patients. **C** Volcano plot illustrating the screening of differential metabolites in urine. **D** Venn diagram showing the overlap of differential metabolites between the discovery and validation cohorts for urine samples. **E** Binary logistic regression analysis of urinary differential metabolites. OR, odds ratio; An OR = 1 indicates that the variable is not associated with the outcome; an OR > 1 suggests that the variable increases the risk of the outcome, whereas an OR < 1 implies that the variable reduces the risk. **F** KEGG pathway enrichment analysis of the differential metabolites in urine. **G**, **H** PCA and UMAP of serum metabolomic data reveal a distinct separation between the HC group and GDHS patients. **I** Venn diagram depicting the overlap of differential molecules between the discovery and validation cohorts for serum samples. **J** Circular heatmap of serum differential molecules. **K** KEGG pathway enrichment analysis of the differential metabolites in serum

To systematically characterize the metabolic remodeling in GDHS, we conducted metabolomic analyses of serum and urine samples from both GDHS patients and HC individuals, and performed visualization using multiple dimensionality reduction techniques. PCA was first employed as an exploratory tool to assess overall trends in metabolic/ lipoprotein profile disparities between groups (Fig. 2A, G). To capture complex nonlinear structures within the high-dimensional data, we subsequently applied t-SNE (Fig. 2B) and UMAP (Fig. 2H) for in-depth visualization. These methods are complementary: PCA reveals linear features along directions of maximum variance, t-SNE emphasizes the preservation of local similarity structures, and UMAP excels in balancing both global and local topological relationships [41–43]. All three approaches consistently demonstrated a clear separation trend between GDHS and HC samples in low-dimensional space, collectively indicating significant metabolic reprogramming in GDHS patients.

Subsequently, differentially expressed features were initially identified using a combined threshold of |log₂ (FC)|> 0.26 (equivalent to FC > 1.2 or < 0.83) and a P < 0.05 derived from univariate statistical analysis. To minimize false positives and enhance the robustness of the findings, we further excluded features that showed inconsistent trends between the discovery and validation cohorts or were significant in only one cohort (Fig. S1). Ultimately, 12 differential urinary metabolites (Fig. 2C–E, Table S3) and 70 differential serum metabolites/ lipoproteins/ inflammatory factors (Fig. 2I, J, Table S3) were screened. Notably, the vast majority of significant differential molecules originated from serum, whereas urinary metabolites exhibited considerably fewer alterations, suggesting that the serum metabolome may possess higher sensitivity and greater informational richness in reflecting GDHS disease status.

To further elucidate the biological mechanisms underlying GDHS, we performed pathway enrichment analysis using the MetaboAnalyst platform (Fig. 2F, K, Table S4). The results (Impact > 0.1, P < 0.05) indicated that eight metabolic pathways were significantly perturbed in serum: glyoxylate and dicarboxylate metabolism; alanine, aspartate and glutamate metabolism; the citrate cycle; pyruvate metabolism; glycolysis or gluconeogenesis; one-carbon pool by folate; glycine, serine and threonine metabolism; and cysteine and methionine metabolism. In urine, four pathways were significantly perturbed: glycine, serine and threonine metabolism; glyoxylate and dicarboxylate metabolism; arginine and proline metabolism; and phenylalanine metabolism.

### A unique dyslipidemia pattern in GDHS patients: characterized by VLDL-IDL metabolic defects and HDL reduction

**Fig. 3:**
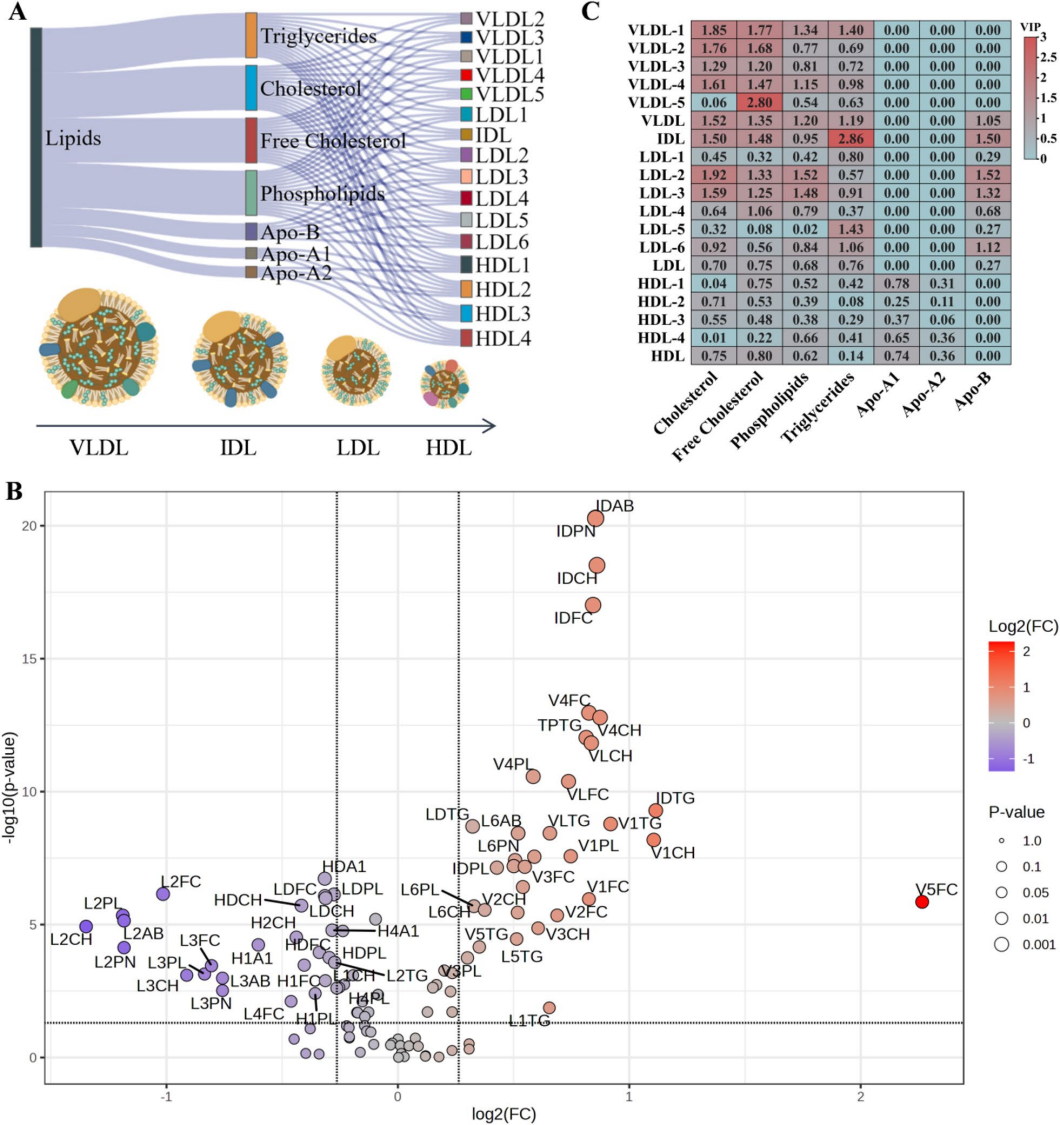
Imbalance of lipoprotein subfractions in GDHS patients. **A** The 112 lipoprotein subfractions in this study; **B** Volcano plot of lipoprotein subfractions (Significance threshold: P < 0.05 and |log₂FC|> 0.263). Red dots represent lipoprotein subfractions significantly elevated in GDHS patients, while blue dots represent those significantly reduced. **C** Heatmap of lipoprotein subfractions based on Variable Importance in Projection (VIP) analysis. The intensity of the red color corresponds to the contribution of each subfraction in discriminating between GDHS patients and HC individuals. *TG* Triglycerides, *CH* Cholesterol, *FC* Free cholesterol, *PL* Phospholipids, *A1* Apolipoprotein A1, *A2* Apolipoprotein A2, *AB* Apolipoprotein B

Analysis of lipoprotein subclasses revealed extensive dyslipidemia in GDHS patients, characterized by significantly elevated levels of VLDL and IDL, along with markedly reduced levels of most HDL and LDL subclasses (Fig. 3A–C). This constellation of changes collectively constitutes a distinct lipoprotein profile specific to this syndrome pattern.

**Fig. 4:**
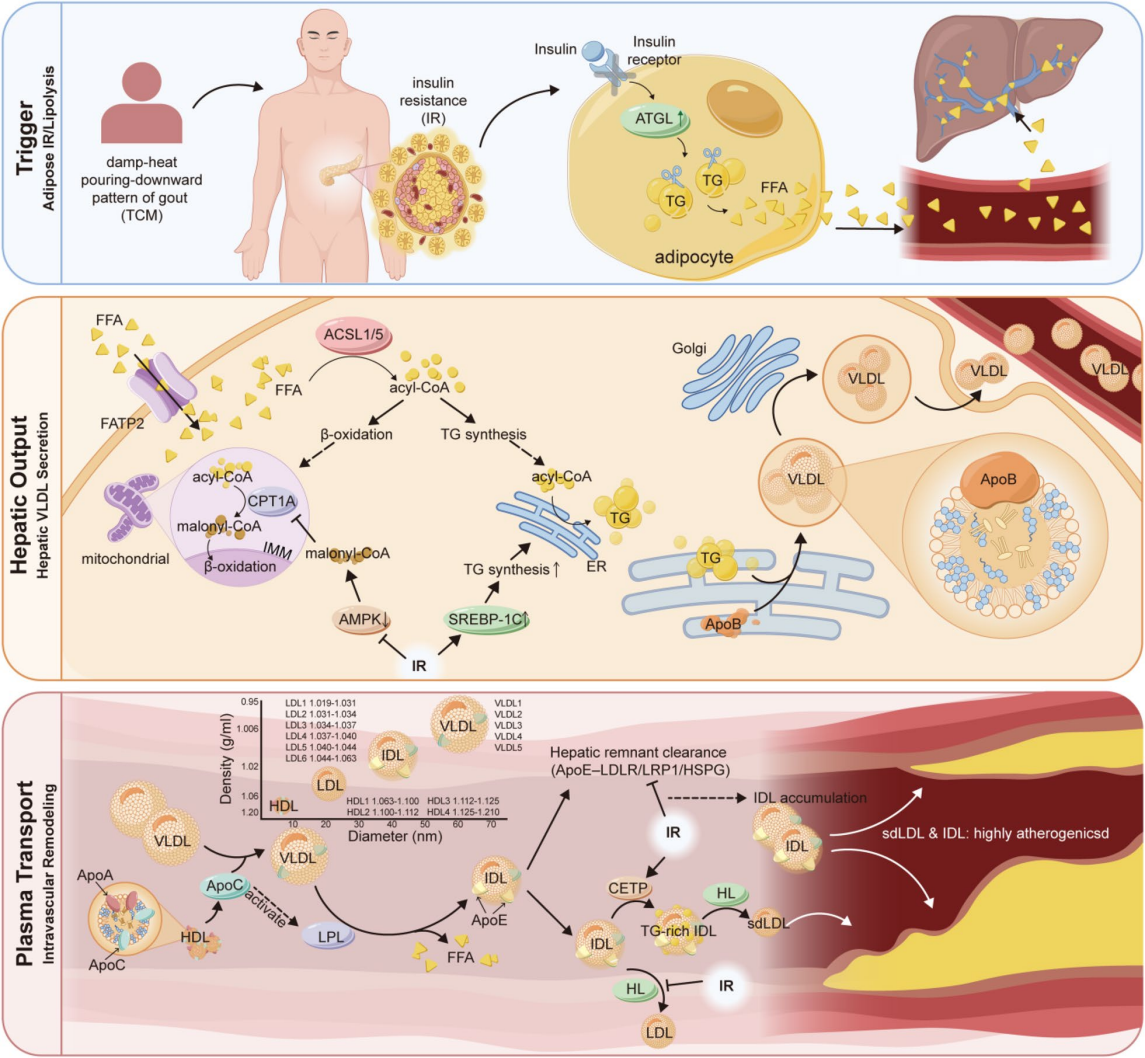
Mechanism of lipoprotein imbalance in GDHS patients. The schematic illustrates the pathological process in three layers: The triggering layer: Insulin resistance and free fatty acid overflow; The hepatic output Layer: Suppressed β-Oxidation and excessive VLDL secretion; The Blood transport layer: Impaired remodeling and atherogenic lipoprotein accumulation. *IR* insulin resistance, *FFA* free fatty acids, *TG* triglyceride, *ATGL* adipose triglyceride lipase, *FATP2* fatty acid transport protein 2, *ACSL1/5* acyl-CoA synthetase long-chain family member 1 and 5, *acyl-CoA* acyl-coenzyme A, *AMPK* AMP-activated protein kinase, *SREBP-1c* sterol regulatory element-binding protein 1c, *CPT1A* carnitine palmitoyltransferase 1A, *ER* endoplasmic reticulum, *RER* rough endoplasmic reticulum, *Apo* apolipoprotein, *LPL* lipoprotein lipase, *HL* hepatic lipase, *EL* endothelial lipase, *CETP* cholesteryl ester transfer protein, *LDLR* low-density lipoprotein receptor, *LRP1* LDL receptor-related protein 1, *HSPG* heparan sulfate proteoglycans, *PCSK9* proprotein convertase subtilisin/kexin type 9

This metabolic phenotype is closely linked to IR and heightened hepatic lipogenesis (Fig. 4). Under IR conditions, hepatic β-oxidation of free fatty acids (FFA) is suppressed, diverting a greater proportion of FFAs toward triglyceride (TG) synthesis. This shift directly drives the production and secretion of TG-rich VLDL particles [44–46]. The overall elevation in VLDL subclasses observed in this study directly reflects this state of enhanced hepatic lipid synthesis. Concurrently, IR promotes uric acid production and inhibits its renal excretion [39]. Thus, hyper-VLDL-emia and hyperuricemia can be considered pathogenically homologous, collectively forming the key characteristics of GDHS.

IDL, an intermediate in the conversion of VLDL to LDL [47–49], accumulates when lipoprotein lipase-mediated VLDL hydrolysis is impaired. Our observation of IDL subclass accumulation indicates that a substantial number of VLDL particles are hydrolyzed but remain at the IDL stage, failing to convert efficiently into typical LDL. Since these retained IDL remnants are small, cholesterol-rich, and highly atherogenic, our findings offer a novel perspective for elucidating the mechanism underlying the increased risk of atherosclerotic cardiovascular disease (ASCVD) in GDHS patients [50–52].

The observed reduction in HDL and its subclasses aligns with established evidence. The core function of HDL is reverse cholesterol transport, conferring anti-inflammatory and antioxidant effects [53–55]. Under conditions of IR and chronic inflammation, however, HDL turnover is accelerated and its synthesis reduced. This impairment of protective functions aligns with the persistent inflammatory state and elevated cardiovascular risk characteristic of this patient population [56].

Our results demonstrated a reduction in most LDL subclasses–a seemingly paradoxical yet critical and valuable finding. This decrease should not be interpreted as a reduced risk, but rather reflects severe dyslipidemia and a dynamic shift in lipid metabolism. On one hand, impaired conversion of VLDL to IDL results in insufficient "raw material" for the generation of canonical LDL particles. On the other hand, under systemic inflammation, LDL particles are more susceptible to clearance by macrophages via scavenger receptor pathways, leading to a shortened circulation time and consequently reduced concentration [57–59]. This suggests that conventional LDL-C likely underestimates cardiovascular risk in these patients, underscoring the need for clinical practice to incorporate markers such as high IDL and low HDL, or to utilize more integrated metrics like non-HDL-C and ApoB, for a comprehensive risk assessment and management strategy.

In summary, this lipoprotein profile forms a characteristic fingerprint of GDHS. It not only reflects disordered hepatic lipid metabolism but also suggests its potential as a novel benchmark for ASCVD risk assessment in these patients. Of course, the exact mechanism behind this unique phenomenon still needs further clarification through subsequent research.

### A robust 12-biomarker panel with diagnostic value for GDHS

**Fig. 5:**
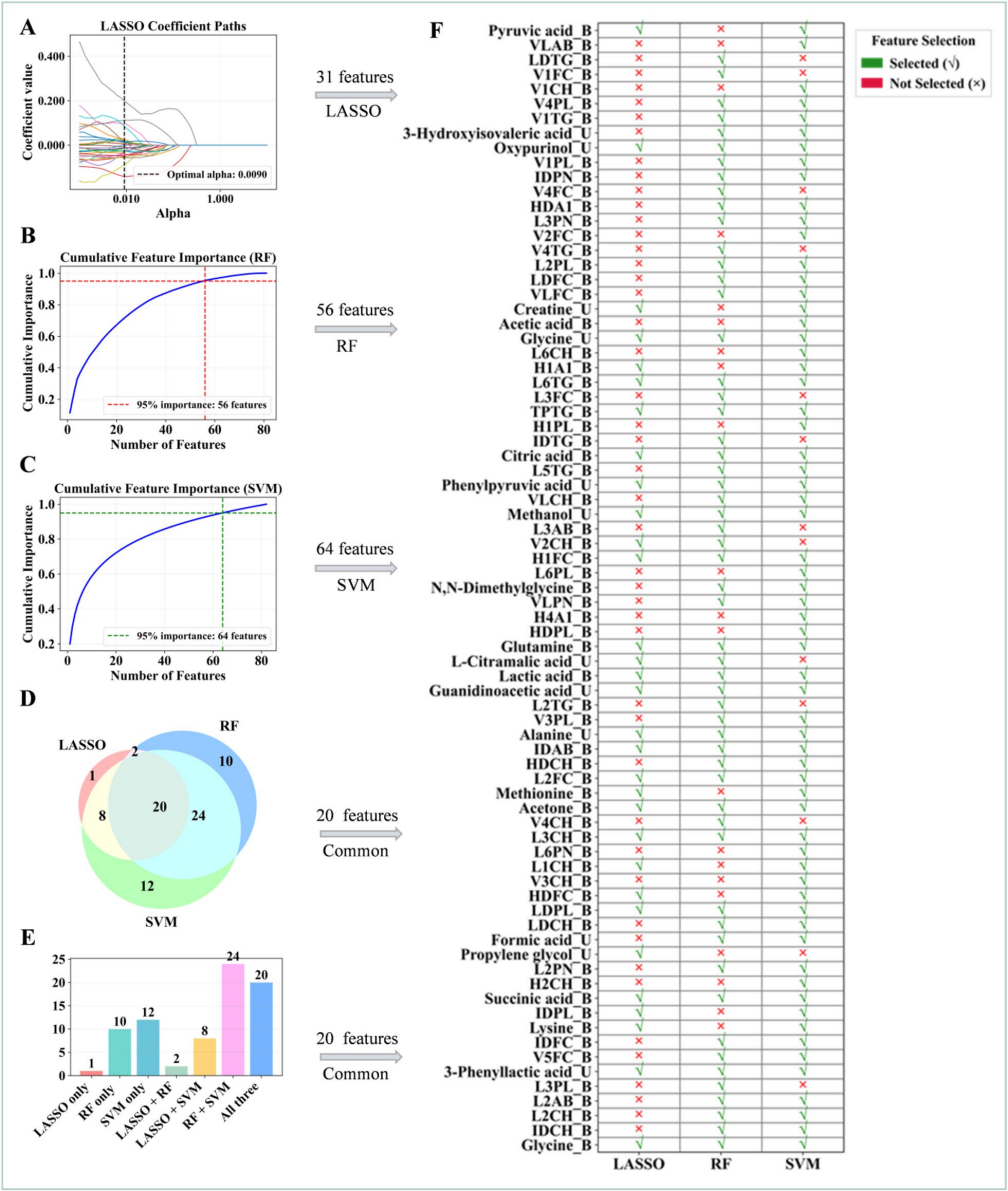
Screening of potential biomarkers for GDHS. **A** 31 important features were selected using the least absolute shrinkage and selection operator (LASSO) regression algorithm. **B** 56 important features were identified by the Random Forest (RF) algorithm. **C** 64 important features were selected via the Support Vector Machine (SVM) algorithm. **D**, **E** The overlapping features from the three algorithms were defined as potential biomarkers for GDHS. **F** Visualization of the important features selected by the three respective algorithms

**Fig. 6:**
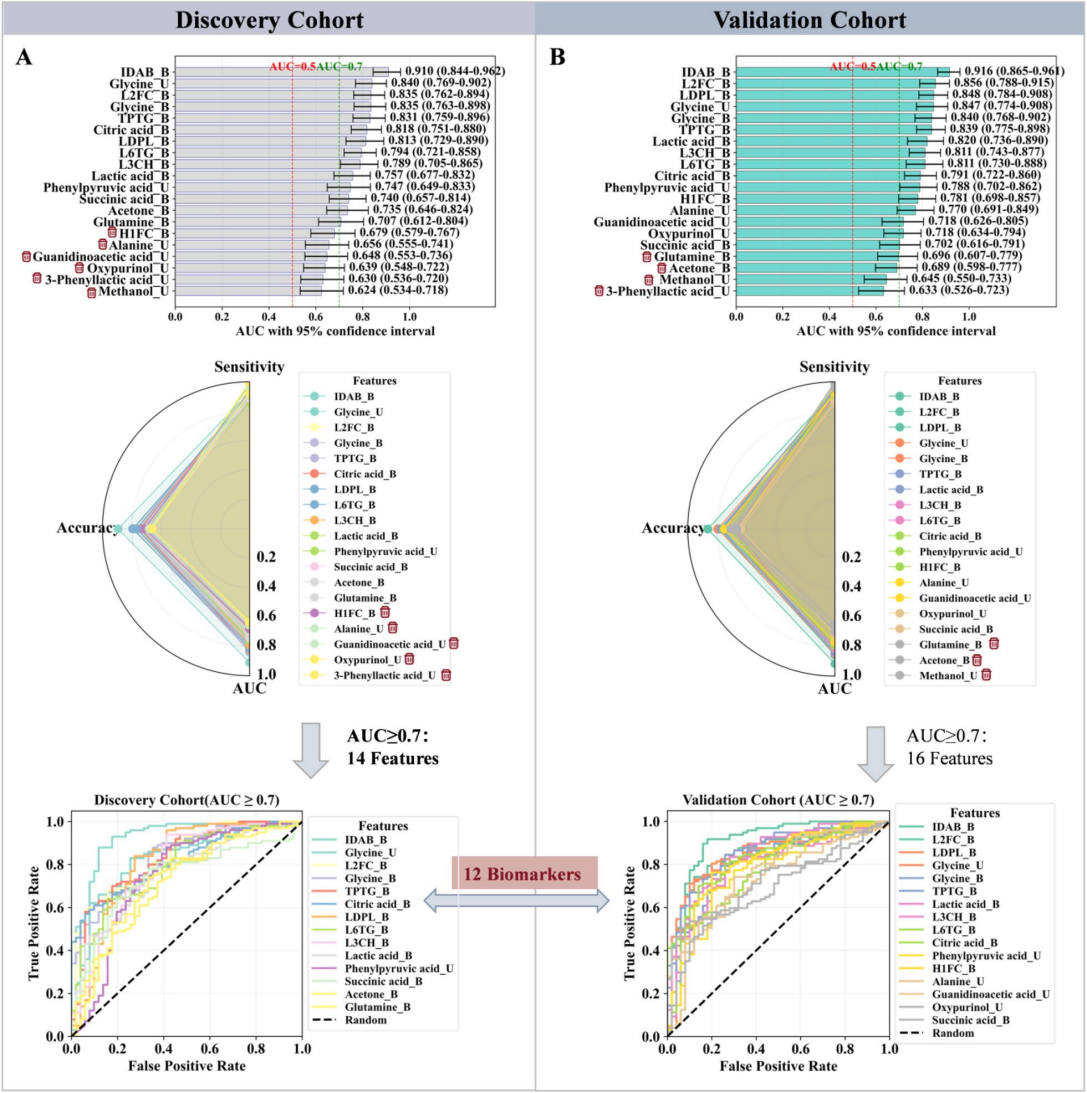
Diagnostic performance evaluation of potential biomarkers for GDHS. **A** Evaluation based on the discovery cohort. **B** Evaluation based on the validation cohort. Only the features that demonstrated consistently good performance in both cohorts were ultimately identified as biomarkers for GDHS

This study employed a multi-algorithm integration strategy to identify biomarkers for GDHS. In the discovery cohort, we applied three complementary machine learning algorithms: LASSO regression screened 31 key features (Fig. 5A), RF determined 56 core features (Fig. 5B), and SVM-RFE selected 64 key features (Fig. 5C). To enhance the robustness of the biomarkers, we extracted the intersection of features commonly seleced by all three algorithms, resulting in 20 consistently present candidate biomarkers (Fig. 5D–F). The diagnostic performance of these candidate biomarkers was further evaluated using ROC curve analysis, which comprehensively assesses the trade-off between sensitivity and specificity across different thresholds, with the AUC providing a robust measure of overall accuracy. Ultimately, 12 features that exhibited stable discriminative performance (AUC ≥ 0.7) in both cohorts were selected as biomarkers of GDHS (Fig. 6). The panel comprises IDAB, L2FC, TPTG, LDPL, L6TG, L3CH, glycine, citric acid, lactic acid, and succinic acid in serum, along with phenylpyruvic acid and glycine in urine (Table S5, S6, Fig. S2, S3). These biomarkers span multiple core metabolic processes of GDHS. Specifically, glycine serves as a pivotal molecule in glyoxylate/dicarboxylate, one-carbon, and glycine/serine/threonine metabolism. Citrate and succinate, as central intermediates of the TCA cycle, reflect the functional state of the energy metabolism hub and indicate potential disturbances in closely linked amino acid metabolic pathways. Lactate, a direct participant in pyruvate metabolism and glycolysis/gluconeogenesis, mirrors alterations in glycolytic flux. Phenylpyruvate is a key intermediate in phenylalanine metabolism. Meanwhile, lipoprotein subclasses such as IDAB, L2FC, and TPTG signify an imbalance in lipid metabolic homeostasis.

In conclusion, this strategy effectively leveraged the strengths of distinct algorithms and, combined with multi-cohort validation, significantly enhanced the reliability of the screening results, thereby providing a system of biologically meaningful key molecular targets for subsequent research.

### A biomarker-syndrome network in GDHS with triglycerides and glycine as central hubs

**Fig. 7:**
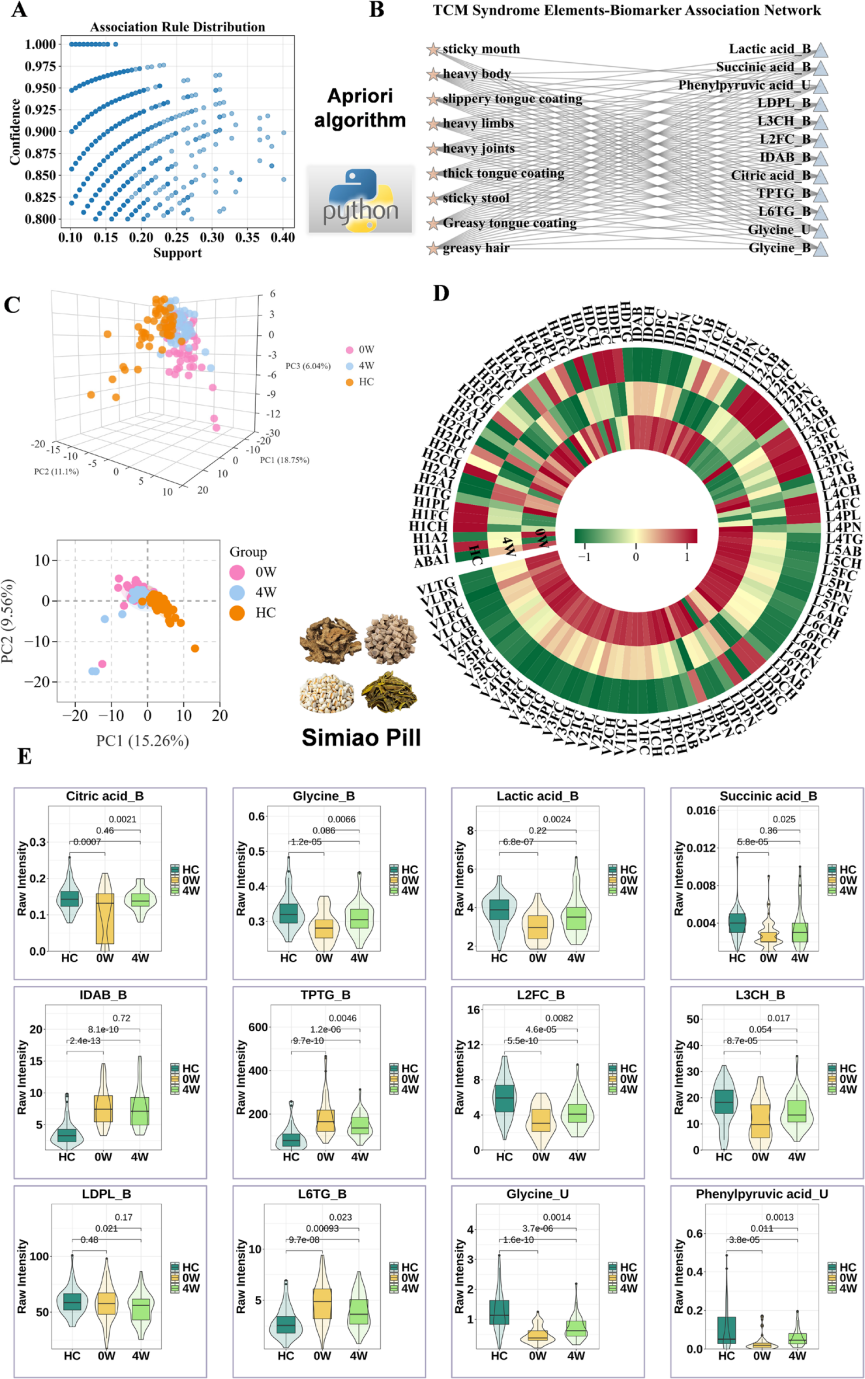
Association of TCM syndrome elements with biomarkers and the therapeutic effect of Simiao Pill on GDHS. **A**, **B** Results of the correlation analysis(via Apriori algorithm). **C** Regulatory effect of Simiao Pills on the metabolic profile of GDHS patients. **D** Impact of SMP on the Imbalanced Lipoprotein Profile in GDHS Patients. **E** Simiao Pills modulating the biomarker levels

Association rule mining successfully uncovered a complex co-occurrence network between serum/urinary biomarkers and core syndrome elements in GDHS, systematically mapping the relationships between macroscopic symptoms and microscopic molecules (Fig. 7A, B, Table S7). Notably, the analysis revealed significant biomarker associations for nine of the eleven major TCM syndrome elements, whereas no strong rules were identified for "heavy head sensation" and "scrotal dampness" under the set thresholds. This observation may be influenced by multiple factors, including the coverage of the selected biomarker panel, the technical characteristics of the detection platform, and the settings of the algorithm parameters. A prominent finding was the central role of triglycerides. TPTG and L6TG demonstrated the highest network centrality, being associated with all 9 of the other syndrome elements (with a maximum lift of 4.259), strongly suggesting a close co-occurrence relationship between lipid metabolism disorders and the broad clinical manifestations of GDHS. Somatic symptoms in these patients were characterized by a "heavy" sensation. "Heavy body","heavy limbs" and "heavy joints" were all embedded within a background of extensive lipid and energy metabolism disturbances. Among these, "heavy joints" was specifically and strongly associated with lactic acid. This difference in association patterns provides molecular clues for understanding the subtle pathological mechanisms underlying the "heavy sensation" in different body regions. Furthermore, the scope of glycine's associations encompassed various clinical manifestations, ranging from somatic sensations (e.g., heavy body), tongue features (e.g., thick, greasy coating), to secretions (e.g., greasy hair). This extensive co-occurrence pattern indicates that glycine abnormality is linked to the majority of macroscopic symptoms in GDHS. Our findings also provides metabolomic clues for TCM pathological concept of "sticky and stagnant turbid dampness". "Sticky mouth" was associated with a relatively refined set of biomarkers (e.g., glycine, phenylpyruvic acid), whereas "sloppy, sticky stool" was associated with the entire set of 12 biomarkers. Additionally, the tongue coating, a crucial diagnostic indicator in TCM, demonstrated highly valuable metabolic correlations: the "greasy tongue coating" was widely associated with various levels of all 12 biomarkers; the association network for the "thick tongue coating" was relatively concentrated, with the association to glycine being particularly prominent; and the "smooth and watery tongue coating" was primarily associated with specific lipid molecules like LDPL and L2FC. The distinct association patterns among these three tongue coatings provide preliminary evidence for differentiating their microscopic correlates.

### SMP reverses the GDHS metabolic phenotype by rescuing core metabolic pathways

After a standardized 4-week therapy with SMP, PCA analysis demonstrated a significant shift in the post-treatment metabolic profile toward the HC group, indicating that SMP effectively reversed the systemic metabolic disturbances induced by GDHS (Fig. 7C). Further analysis showed that SMP intervention significantly reversed the characteristic lipoprotein abnormality profile of GDHS (Fig. 7D). Given its link to IR, we propose that the remedy alleviates IR, thereby suppressing hepatic lipogenesis, reducing VLDL output, and potentially modulating key lipoprotein metabolic enzymes. This coordinated action restores lipoprotein homeostasis, with important implications for reducing cardiovascular comorbidity risk in these patients. Quantitative validation of the 12 previously identified specific biomarkers showed that 10 were significantly reversed post-intervention, including serum L2FC, TPTG, L6TG, L3CH, glycine, citric acid, lactic acid, and succinic acid, as well as urinary phenylpyruvic acid and glycine (Fig. 7E). The reversal of these biomarkers implicated several key metabolic pathways: the restoration of lactate levels suggested improved glycolysis/gluconeogenesis flux; the modulation of citrate and succinate levels reflected a correction of the tricarboxylic acid cycle disruption; and the adjustment of glycine levels was closely linked to the normalization of one-carbon metabolism and the glycine, serine, and threonine metabolism pathway.

In summary, this study systematically elucidates across three levels—the overall metabolic profile, lipoprotein subclass distribution, and specific biomarkers—that SMP may employ multi-target synergistic actions to regulate core pathways including energy, amino acid, and lipid metabolism. This concerted action ultimately reverses the metabolic reprogramming and characteristic dyslipidemia in the GDHS state, thus reestablishing systemic metabolic homeostasis.

## Discussion

According to a global epidemiologic report of gout in 2020, the prevalence and incidence of gout respectively range from < 1 to 6.8% and 0.58 to 2.89 per 1000 person-years [60], and the mortality of gout may increase by 55% in 2060 [61], positioning gout an increasingly serious public health concern. Current research on gout primarily focuses on uric acid metabolism from the perspective of Western medicine, while exploration into the modern scientific basis of its TCM syndrome patterns remains limited. In this study, we utilized high-precision targeted NMR metabolomics integrated with clinical cohort analysis to systematically characterize the metabolic disturbance pattern in GDHS patients. Through innovative association rule mining, we further revealed intrinsic connections between macroscopic symptoms and microscopic metabolites, providing previously unavailable objective evidence for the scientific basis of this TCM syndrome. Notably, this study established a comprehensive logical framework from pathological mechanism elucidation to therapeutic mechanism verification. This framework not only provides an objective basis for GDHS diagnosis but also establishes a target system for validating SMP's mechanism of action.

The pathway enrichment analysis results indicate that the metabolic remodeling in GDHS is primarily reflected in perturbations of core energy metabolism and amino acid metabolic pathways. Among these, glyoxylate and dicarboxylate metabolism, and glycine, serine, and threonine metabolism were commonly enriched in both sample types, suggesting their pivotal roles in GDHS pathogenesis. The glyoxylate and dicarboxylate metabolism pathway is tightly integrated with the TCA cycle through shared key intermediate metabolites. Perturbations in this pathway can directly disrupt TCA cycle homeostasis, as indicated by altered levels of citrate, succinate, and pyruvate, which collectively reveal anomalies in energy metabolic flux. This dysregulation may lead to the accumulation of pivotal precursors such as acetyl-CoA and oxaloacetate, ultimately raising phosphoribosyl pyrophosphate levels and thereby supplying excessive substrate for de novo purine synthesis [62–64]. This mechanism may represent a potential driver of excessive endogenous uric acid production. In parallel, disruption of glycine, serine, and threonine metabolism contribute to disease progression through a dual mechanism. First, this pathway directly supplies molecular backbones such as glycine, essential for purine synthesis, and synergizes with one‑carbon metabolism, thereby sustaining a hyperactive state of purine metabolism [65, 66]. Second, glycine and glutamine serve as key substrates for the synthesis of glutathione, a major antioxidant. Their metabolic dysregulation inevitably impairs the body’s antioxidant defense capacity [67–69]. This impairment aligns with the chronic inflammatory state commonly observed in GDHS. Collectively, these findings reveal a pathological network characterized by aberrant energy metabolism, enhanced purine synthesis, and oxidative stress, which systemically drives the onset and progression of GDHS.

Additionally, the unique lipoprotein imbalance pattern identified in this study offers novel insights into the elevated cardiovascular comorbidity risk in GDHS patients. This pattern is closely associated with IR: under IR conditions, excess FFA flood the liver, shifting metabolic focus from β-oxidation to TG synthesis and VLDL assembly, directly leading to high VLDL levels [70–72]. IDL accumulation suggests impaired clearance of TG-rich lipoprotein remnants, potentially related to reduced efficiency of the heparan sulfate proteoglycan/hepatic lipase (HSPG/HL) platform and proprotein convertase subtilisin/kexin type 9 (PCSK9)-mediated receptor dysfunction [73–75]. The decrease in HDL may involve cholesteryl ester transfer protein (CETP)-mediated lipid exchange and subsequent hydrolysis, reflecting compromised reverse cholesterol transport [76–78]. The reduction in most LDL subclasses likely results from both upstream VLDL-IDL metabolic flow obstruction and accelerated clearance in an inflammatory environment [57–59]. Therefore, lipid management in GDHS patients should focus on correcting dysregulation of the entire lipoprotein metabolic system rather than targeting individual parameters.

The mechanisms underlying "heaviness" and "stickiness", the hallmark manifestations of this TCM syndrome, were preliminarily elucidated. The specific association between lactate and "heavy joints" suggests that energy metabolism disturbances may contribute to this symptom. Lactate might directly mediate abnormal sensory signaling related to the "heaviness" sensation by activating acid-sensing ion channels (e.g., ASIC3, TRPV1) on peripheral sensory nerves [79, 80]. Additionally, aberrant glycine levels could potentially exacerbate this sensation by enhancing inhibitory neural signals through effects on glycine receptor (GlyR) function [81, 82]. The "stickiness" symptoms reflect localized manifestations of systemic metabolic disturbances at mucosal sites. The extensive associations of "sticky stool" indicate potential significant gut microbiota-host co-metabolism dysregulation. For instance, aberrant succinate levels may influence the intestinal environment via the SUCNR1 signaling pathway, while abnormal phenylpyruvic acid suggests a potential impact of aromatic amino acid metabolism dysregulation on the gut microecology [80, 83, 84]. In contrast, the more focused metabolic signature of "sticky mouth" suggests a mechanism more specific to the oral microenvironment and salivary composition changes. Furthermore, distinct metabolic networks corresponding to different tongue coatings (thick, greasy, moist) provide a basis for modern interpretation of TCM tongue diagnosis.

While our study reveals the therapeutic effect of SMP in GDHS from a metabolic regulation perspective, its precise pharmacodynamic material basis remains incompletely elucidated. Existing research on Sanmiao Pill suggests potential active components including atractylenolide III, atractylenolide II, achyranthanoside C, geniposide, and berberine [85]. Given that SMP is formulated by adding Coicis Semen to Sanmiao Pill, these findings may offer valuable clues for identifying the pharmacodynamic material basis of SMP.

In summary, this study systematically characterized the metabolic profile of GDHS using targeted metabolomics and multiple machine learning algorithms, revealed the holistic regulatory effect of SMP, and established a feasible paradigm for TCM research on "disease‑syndrome‑treatment". However, several limitations should be acknowledged. First, although the sample size was sufficient for preliminary exploration, the single-center cohort with relatively limited scale may affect the generalizability of our findings. Future studies with larger, multi-center cohorts are needed to validate and extend these discoveries. Second, while the "syndrome-biomarker" network constructed through association rule mining represents robust correlations derived from high-dimensional data, the biological mechanisms underlying these associations require further validation using animal models. Finally, although SMP demonstrated overall regulatory effects on the metabolic network, their precise active components, in vivo metabolic processes, and specific molecular targets downstream remain incompletely elucidated. Future research should integrate pharmacokinetic and molecular biological approaches to systematically investigate these aspects across multiple levels, from systems to cells to molecules.

## Conclusion

The study elucidates the distinct metabolic remodeling in GDHS patients, identifying a panel of biomarkers with strong diagnostic potential—including serum IDAB, L2FC, TPTG, LDPL, L6TG, L3CH, glycine, citric acid, lactic acid, and succinic acid, as well as urinary phenylpyruvic acid and glycine. Furthermore, association rule mining revealed extensive interactions between core molecules, such as triglycerides and glycine, and TCM syndrome elements, thereby providing novel insights into the scientific basis of GDHS. Finally, we demonstrated the efficacy of SMP in reversing the identified metabolic disturbances, supporting its broader clinical application.

## Supplementary Information

Supplementary material 1.

Supplementary material 2.

## Data Availability

The datasets used and/or analysed during the current study are available from the corresponding author on reasonable request.

## Abbreviations

AUC: Area under the curve
BMI: Body mass index
CETP: Cholesteryl ester transfer protein
CH: Cholesterol
CPMG: Carr–Purcell–Meibo-Gill
FC: Free cholesterol
FFA: Free fatty acids
FID: Free induction decay
GDHS: Gout with the dampness-heat syndrome
GlyR: Glycine recepto
HC: Healthy control
HDL: High-density lipoprotein
HSPG/HL: Heparan sulfate proteoglycan/hepatic lipase
IDL: Intermediate-density lipoprotein
IR: Insulin-resistant
IVDr: In vitro diagnostic research
KEGG: Kyoto encyclopedia of genes and genomes
LASSO: Least absolute shrinkage and selection operator
LDL: Low-density lipoprotein
NMR: Nuclear magnetic resonance
NOESY: Nuclear overhauser enhancement spectroscopy
PCA: Principal component analysis
PCSK9: Proprotein convertase subtilisin/kexin type 9
PL: Phospholipids
RF: Random forest
ROC: Receiver operating characteristic
SMP: Simiao Pill
SVM: Support vector machine
TCM: Traditional Chinese medicine
TG: Triglyceride
t-SNE: T-distributed stochastic neighbor embedding
UMAP: Uniform manifold approximation and projection
VLDL: Very low-density lipoprotein
