# Supplementary material for: NMR-based metabolomics in a clinical cohort: deciphering the metabolic characteristics of gout with the dampness-heat syndrome and elucidate the efficacy of Simiao Pill
Source: Chin Med. 2026 Jan 21;21:43. doi: 10.1186/s13020-025-01289-6 (PMC12822329; doi:10.1186/s13020-025-01289-6)
Supplement: Supplementary file 2 — Supplementary material 2. [file 13020_2025_1289_MOESM2_ESM.docx]

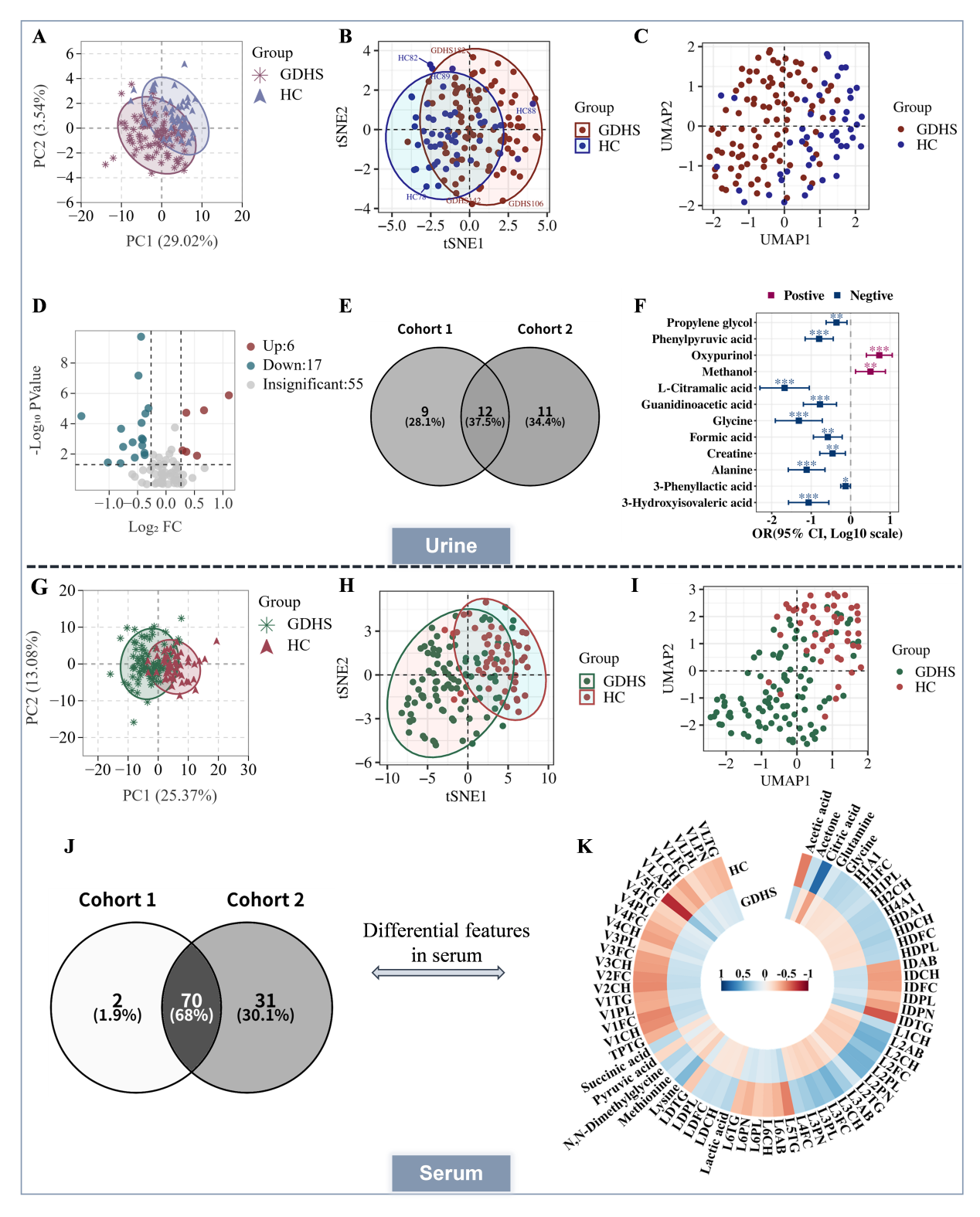


**Fig. S1** Metabolic reprogramming in GDHS patients (validation cohort). **A-C** PCA, t-SNE and UMAP of urinary metabolomic data demonstrate distinct separation between the HC group and GDHS patients. **D** Volcano plot illustrating the screening of differential metabolites in urine. **E** Venn diagram showing the overlap of differential metabolites between the discovery and validation cohorts for urine samples. **F** Binary logistic regression analysis of urinary differential metabolites. OR, odds ratio; An OR = 1 indicates that the variable is not associated with the outcome; an OR > 1 suggests that the variable increases the risk of the outcome, whereas an OR < 1 implies that the variable reduces the risk. **G-I** PCA, t-SNE and UMAP of serum metabolomic data reveal a distinct separation between the HC group and GDHS patients. **J** Venn diagram depicting the overlap of differential molecules between the discovery and validation cohorts for serum samples. **K** Circular heatmap of serum differential molecules.


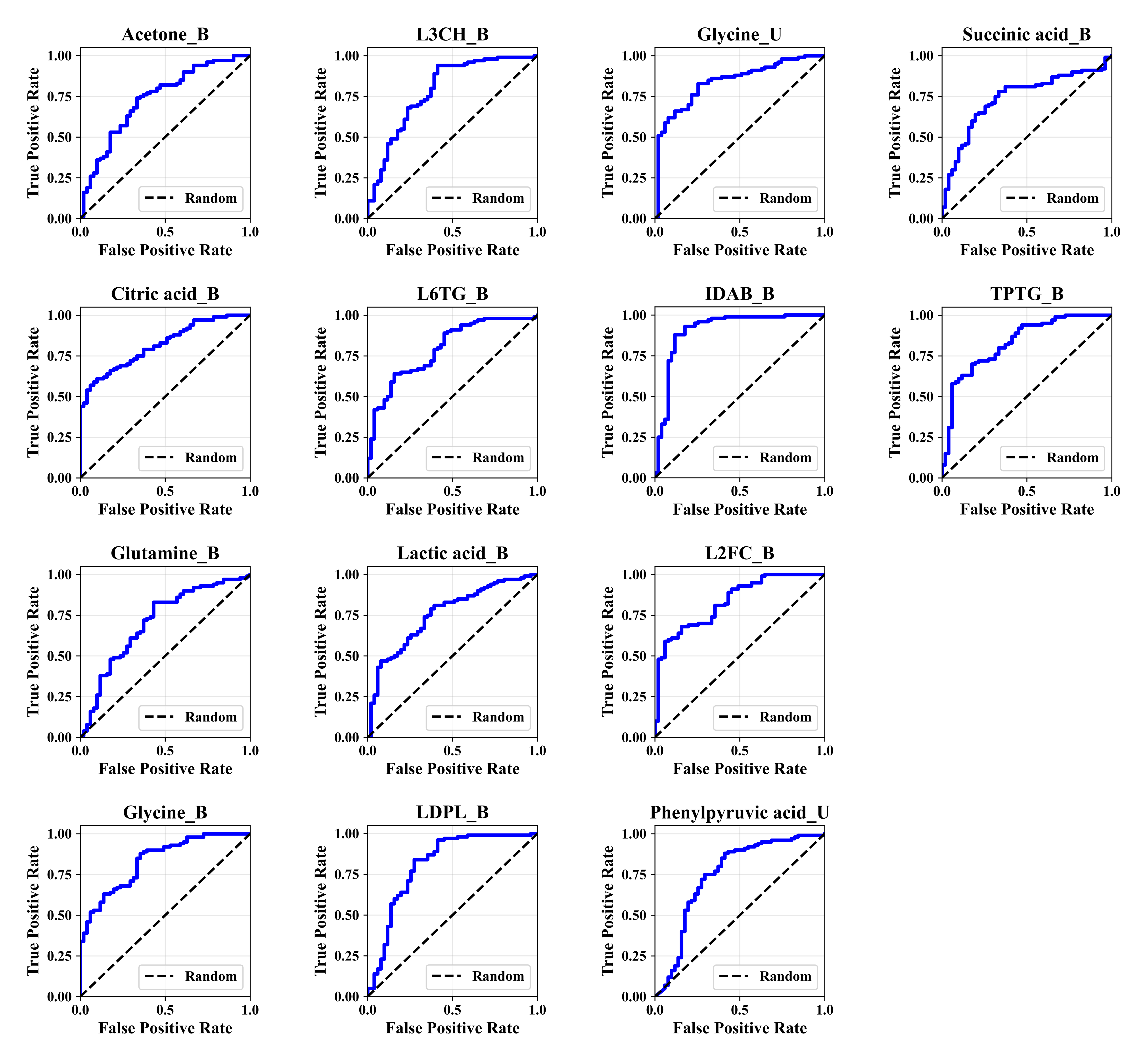


**Fig. S2** Potential biomarkers with AUC > 0.7 in the discovery cohort. Those maintaining an AUC > 0.7 in both discovery and validation cohorts were ultimately defined as biomarkers for GDHS.


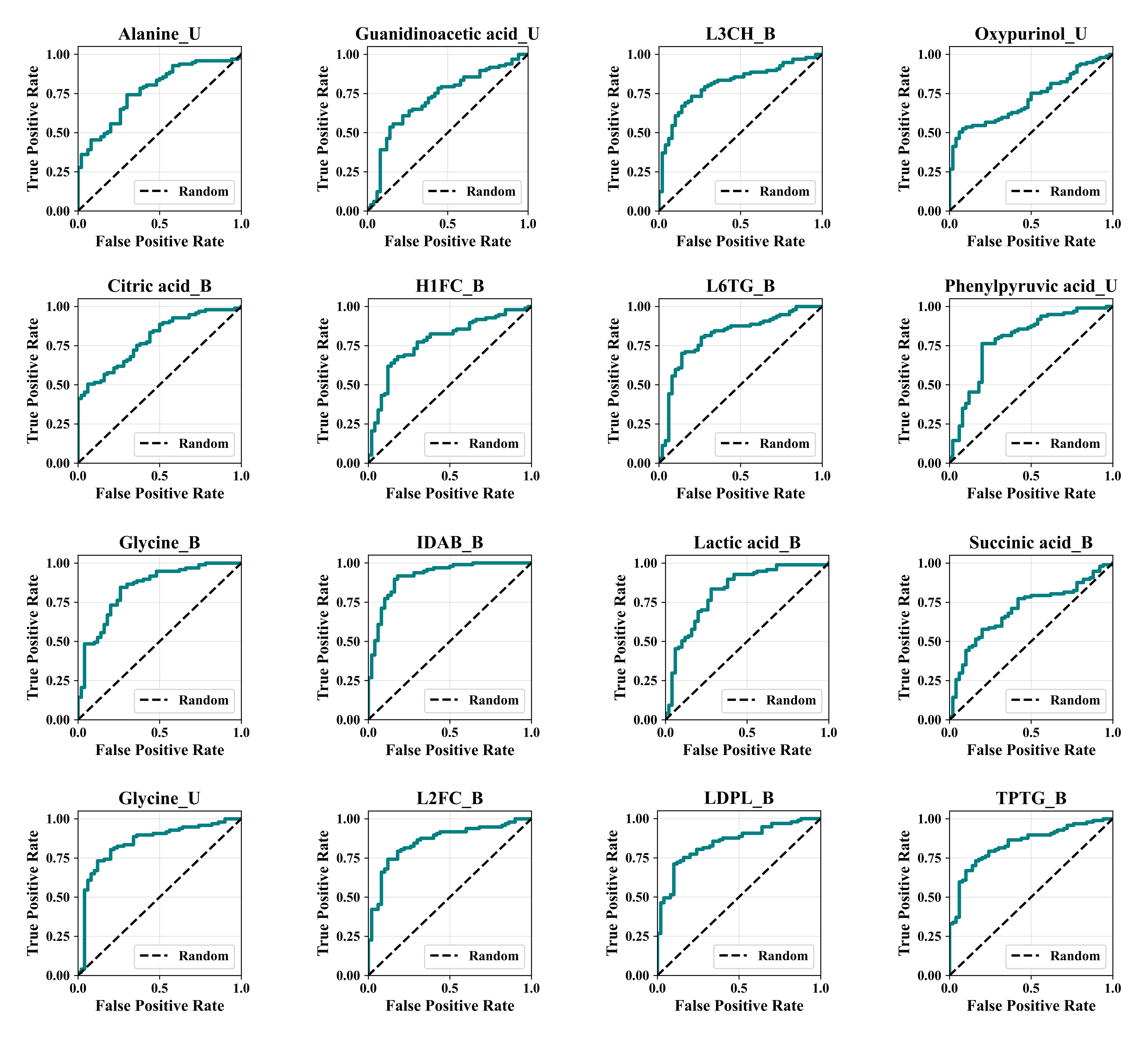


**Fig. S3** Potential biomarkers with AUC > 0.7 in the validation cohort. Those maintaining an AUC > 0.7 in both discovery and validation cohorts were ultimately defined as biomarkers for GDHS.
